# Auxiliary Truncated Unscented Kalman Filtering for Bearings-Only Maneuvering Target Tracking

**DOI:** 10.3390/s17050972

**Published:** 2017-04-27

**Authors:** Liang-Qun Li, Xiao-Li Wang, Zong-Xiang Liu, Wei-Xin Xie

**Affiliations:** Automatic Target Recognition Key Laboratory (ATR), Shenzhen University, Shenzhen 518060, China; wangxiaoli2016@email.szu.edu.cn (X.-L.W.); liuzx@szu.edu.cn (Z.-X.L.); wxxie@szu.edu.cn (W.-X.X.)

**Keywords:** bearings-only target tracking, statistical linear regression, auxiliary truncated unscented Kalman filtering

## Abstract

Novel auxiliary truncated unscented Kalman filtering (ATUKF) is proposed for bearings-only maneuvering target tracking in this paper. In the proposed algorithm, to deal with arbitrary changes in motion models, a modified prior probability density function (PDF) is derived based on some auxiliary target characteristics and current measurements. Then, the modified prior PDF is approximated as a Gaussian density by using the statistical linear regression (SLR) to estimate the mean and covariance. In order to track bearings-only maneuvering target, the posterior PDF is jointly estimated based on the prior probability density function and the modified prior probability density function, and a practical algorithm is developed. Finally, compared with other nonlinear filtering approaches, the experimental results of the proposed algorithm show a significant improvement for both the univariate nonstationary growth model (UNGM) case and bearings-only target tracking case.

## 1. Introduction

Bearings-only maneuvering target tracking has been widely researched for decades. It is important for many applications such as maritime surveillance, navigation and aerospace, wireless sensor networks (WSN), and infrared search and track (IRST) systems [1,2,3,4,5,6]. However, while implementing this technology in unlimited situations, there remain some challenging problems, such as multiple platform tracking, uncertainty of the target model and nonlinear non-Gaussian noise. To deal with the uncertainty of the motion model, such as abrupt target maneuver, heavy clutter measurements, highly nonlinearity of dynamic models and nonlinear non-Gaussian noise, etc., the interacting multiple model (IMM) [7] based on the nonlinear filtering algorithm is a promising approach. However, to model the uncertainty of the motion model, the performance of the IMM-type algorithm is directly proportional to the number of the motion models. Generally, the more motion models we produce, the greater accuracy of the estimated state we obtain. However, the computational complexity of the algorithm becomes larger with the increase of the numbers of motion models, particularly in heavily cluttered environments. Moreover, the nonlinear filtering has been studied extensively in bearings-only maneuvering target tracking.

As is well-known, the most widely used nonlinear filtering for bearing-only tracking is to employ an extended Kalman filter (EKF) [8,9]. However, when the nonlinearity of dynamic models becomes more severe, the performance of the EKF degrades sharply. In order to solve this problem, the unscented Kalman filter (UKF) [10] and the truncated unscented Kalman filtering (TUKF) were proposed [11,12]. Compared with other conventional Kalman filter-type approaches, the TUKF can achieve better performance in the conditions of the target tracking system, and can provide very informative nonlinear measurements compared to the prior. Moreover, to take into account the available additional information the state given by the constraint, Ondrej et al. [13] proposed a generic local filter for the inequality constrained estimation problem, and designed an efficient truncation technique based on the Monte Carlo integration method for the approximation of the state probability density function. Beatriz et al. [14] proposed a constrained dual state and parameter estimation algorithm using a dual Kalman filter (DKF) and a probability density function (PDF) truncation algorithm for analysis of lateral vehicle dynamics.

In recent years, particle filtering has been widely used for bearing-only tracking. In [15], Gordon proposed the first particle filtering algorithm based on the resampling step. The main idea is that the posterior distribution can be approximated by series of random samples with associated weights, and its parameter estimates can be computed by these samples and weights. Therefore, particle filtering can deal with nonlinear non-Gaussian problems in terms of the dynamics and measurements. Recently, many particle filtering methods have been proposed [16,17,18], for example, the extended Kalman particle filter (EKF-PF), unscented particle filter (UPF), and the multivariable feedback particle filter (GPF) [18]. Moreover, for the maneuvering target tracking problem, many particle filters have been proposed based on Markovian switching systems [19,20,21,22]. Boers et al. [19] proposed a interacting multiple model particle filter algorithm (IMM-PF) by combining a mixture of the interacting multiple model (IMM) filter with the particle filter. For the maneuvering target tracking problem in bearings-only wireless sensor networks (WSNs), Atiyeh et al. [20] proposed a interacting multiple model particle filter to estimate the state variables of the moving target. Li et al. [21] proposed a Rao–Blackwellized particle filter based on multiple model algorithm for maneuvering target tracking in a cluttered environment. Yu et al. [22] proposed a distributed particle filter by incorporating the curvature of the sensing region in the measurement model for bearings-only tracking of a moving target. In their method, to reduce the communication load, the transformation of the observations is approximated as Gaussian distribution, which the variance can be approximated using the average variance over all particles. However, abrupt target maneuvers, modeling uncertainty and the high nonlinearity of model function remain to be unsolved issues.

For achieving a successful tracking performance, the aforementioned methods require accurate motion models and adaptive nonlinear filtering methods. However, particularly in maneuvering target tracking, accurate motion modeling is almost impossible, and an adaptive nonlinear filtering needs to be used to handle abrupt maneuver of target. More importantly, these two challenges are not separate problems and should be considered simultaneously. In previous research [23,24], Ehsan et al. [23] proposed a new bearing-only bias estimation model based on triangulation using the associated measurement reports (AMR) or local bearing-only tracks from different sensor pairs for distributed tracking systems. Li et al. [24] proposed novel truncated quadrature Kalman filtering (TQKF) based on the Gauss-Hermite quadrature rule for bearings-only maneuvering target tracking. In order to avoid the requirement of the measurement function being bijective, the modified prior PDF of the TQKF algorithm can be approximately computed by the least square estimation approach. However, the most important limitation of the TQKF is the expensive computational burden, and it cannot be used for real time target tracking.

In this paper, novel auxiliary truncated Kalman filtering (ATUKF) is proposed for bearings-only maneuvering target tracking. Unlike the TUKF algorithm, to overcome the modeling uncertainty, a modified prior probability density function (PDF) is defined based on several auxiliary target characteristics and current measurements, which can effectively minimize the variance of the prior distribution. Moreover, to achieve the requirement of bijective measurement function, the statistical linear regression based on the unscented transformation is used to linearize the nonlinear measurement function, and the modified prior PDF is approximated as Gaussian. Finally, the posterior PDF can be approximately estimated based on the prior PDF and the modified prior PDF, and a practical algorithm is developed for bearings-only target tracking systems.

The rest of the paper is organized as follows. The proposed algorithm is given in Section 2. In Section 3, we provide the experimental results. Finally, some conclusions are given in Section 4.

## 2. Proposed Algorithm

In order to track the maneuvering target, accurate motion modeling and nonlinear filtering are two challenging problems that should not be separated. However, most research on maneuvering tracking investigates these problems separately. In this section, in the bearings-only maneuvering target tracking, novel auxiliary truncated unscented Kalman filtering is proposed. In Section 2.1, the joint prior distribution is approximately constructed. In Section 2.2, in order to track the bearings-only maneuvering target, the modified prior PDF is approximated based on statistical linear regression by introducing the target spatio-temporal information. Section 2.3 summarizes the proposed algorithm.

### 2.1. Joint Prior Distribution

Suppose the target dynamic system can be written as:

(1) $$x_{n}=f(x_{n-1})+m_{n}$$

(2) $$z_{n}=h(x_{n})+e_{n}$$

where $z_{n}\in R^{n_{z}}$ denotes the observation vector at time n , $x_{n}\in R^{n_{x}}$ denotes the target state vector at time n , and f(⋅) and h(⋅) denote the nonlinear state transition function and observation function, respectively.

Suppose that $r_{n}$ denotes the set of target characteristics including c independent components $r_{n}=\{r_{n}^{1},r_{n}^{2},\ldots ,r_{n}^{c}\}$ . In order to derive the proposed algorithm, there are two basic hypotheses, firstly, that the nonlinear function $h_{n}(\cdot )$ in (2) is a bijective, continuous function; and secondly, that the probability density function of the measurement noise $e_{n}$ has bounded, connected support.

(3) $$p_{e_{n}}(v_{n})=0,e_{n}\notin I_{e_{n}}\subset {ℜ}^{n_{z}}$$

where $I_{e_{n}}$ is an $n_{z}$ -dimensional measurement validation region. Therefore, according to the second assumption, the measurement likelihood function can be defined as follows:

(4) $$p(z_{n}|x_{n},r_{n})=p_{e_{n}}(z_{n}-h_{n}(x_{n}))\chi_{I_{e_{n}}}(z_{n}-h_{n}(x_{n}))$$

(5) $$p(z_{n}|x_{n},r_{n})=p_{e_{n}}(z_{n}-h_{n}(x_{n}))\chi_{I_{x_{n}}(z_{n})}(x_{n})$$

(6) $$I_{x_{n}}(z_{n})=\{x_{n}|x_{n}=h_{n}^{-1}(z_{n}-e_{n}),e_{n}\in I_{e_{n}}\}$$

where $\chi_{I_{e_{n}}}(\cdot )$ is the indicator function on the subset $I_{e_{n}}$ . Therefore, the state posterior PDF can be defined as:

(7) $$\begin{array}{ll} p(x_{0:n}|z_{1:n},r_{1:n}) & =\frac{p(z_{n}|x_{0:n},z_{1:n-1},r_{1:n})p(x_{n}|x_{1:n-1},z_{1:n-1},r_{1:n})p(x_{1:n-1}|z_{1:n-1},r_{1:n-1})}{p(z_{n},r_{n}|z_{1:n-1},r_{1:n-1})}\\ & =\frac{p_{e_{k}}(z_{n}-h(x_{n}))\chi_{I_{x}(z_{n})}(x_{n})p(x_{n}|x_{n-1},z_{1:n-1},r_{1:n})p(x_{1:n-1}|z_{1:n-1},r_{1:n-1})}{p(z_{k},r_{k}|z_{1:k-1},r_{1:k-1})}\\ & \propto p(z_{n}|x_{n})p_{1}(x_{n}|z_{n},x_{n-1},r_{1:n})p(x_{1:n-1}|z_{1:n-1},r_{1:n-1}) \end{array}$$

(8) $$p_{1}(x_{n}|z_{n},x_{n-1},r_{1:n})=p(x_{n}|x_{n-1},z_{1:n-1},r_{1:n})\chi_{I_{x_{n}}(z_{n})}(x_{n})/\varepsilon_{1}$$

where $\varepsilon_{1}$ is a constant. From Equation (8), we can see that the modified prior PDF is defined by incorporating the current measurement information $z_{n}$ . According to the conclusions in [11], if the measurement noise is informative, the modified prior $p_{1}(\cdot )$ is not only the minimum variance of the prior $p_{0}(\cdot )$ , but also can improve the algorithm’s performance. Further, to deal with the uncertainty of motion models, the joint prior distribution $p(x_{n}|x_{0:n-1},z_{1:n},r_{1:n})$ of the proposed algorithm can be defined as follows:

(9) $$\begin{array}{ll} p(x_{n}|x_{0:n-1},z_{1:n},r_{1:n}) & =\alpha_{n}p_{1}(x_{n}|z_{n},x_{n-1},r_{1:n})+(1-\alpha_{n})p_{0}(x_{n}|x_{n-1},z_{1:n-1})\\ & =\alpha_{n}p(x_{n}|x_{n-1},r_{1:n})\chi_{I_{x_{n}}(z_{n})}(x_{n})+(1-\alpha_{n})p_{0}(x_{n}|x_{n-1},z_{1:n-1}) \end{array}$$

where $\alpha_{n}\in [0,1]$ is a proper parameter. To approximately calculate the mean and covariance of the posterior distribution, we apply a UKF update to $p_{0}(\cdot )$ (UKF1), and another UKF update to $p_{1}(\cdot )$ (UKF2). Finally, the posterior estimates can be approximately calculated through merging both results obtained by UKF1 and UKF2.

### 2.2. Approximation of $p_{1}(\cdot )$

In the subsection, our object is to approximate the modified prior PDF $p_{1}(\cdot )$ as Gaussian. For this reason, we write the state vector as $x_{n}={\left[{a_{n}}^{T},{b_{n}}^{T}\right]}^{T}$ , where $a_{n}\in {ℜ}^{n_{a}}$ denotes the position components of $x_{n}$ , $b_{n}\in {ℜ}^{n_{b}}$ denotes the velocity components of $x_{n}$ , and $n_{x}=n_{a}+n_{b}$ . The derivation of the mean ${\hat{x}}_{p_{1},n}$ and covariance $P_{p_{1},n}$ of $p_{1}(\cdot )$ is the same as in the truncated unscented Kalman filter (TUKF) [11], which can be shown as follows:

(10) $${\hat{x}}_{p_{1},n}=\left[\begin{array}{l} \mu_{a_{n},1}\\ \mu_{b_{n},1} \end{array}\right],P_{p_{1},n}=\left[\begin{array}{l} \sum_{a_{n},1}\;\sum_{a_{n}b_{n},1}\\ {\left(\sum_{a_{n}b_{n},1}\right)}^{T}\;\sum_{b_{n},1} \end{array}\right]$$

(11) $$\Sigma_{a_{n},1}={\tilde{H}}_{n}^{-1}R_{n}{\left({\tilde{H}}_{n}^{-1}\right)}^{T}$$

where $\mu_{b_{n},1}$ , $\Sigma_{b_{n},1}$ , $\Sigma_{a_{n}b_{n},1}$ can be found in [11]. $\mu_{a_{n},1}$ denotes the estimated mean of state $a_{n}$ , $R_{n}$ denotes the measurement noise covariance, and ${\tilde{H}}_{n}^{-1}={\left[\nabla_{a_{n}}h_{n}^{T}(a_{n})\right]}^{T}{|}_{a_{n}=\mu_{a_{n},1}}$ is the Jacobian of $h_{n}^{T}(a_{n})$ evaluated at $\mu_{a_{n},1}$ .

Now, how to calculate the estimated mean $\mu_{a_{n},1}$ remains a key problem to be solved. For the passive sensor tracking system, the modeling of target dynamic system is a challenging problem when the target maneuvers, and some auxiliary target characteristics need to be used to deal with arbitrary changes in motion models. To achieve a high tracking performance, a statistical linear regression method (SLR) [25] is proposed to estimate the state mean $\mu_{a_{n},1}$ .

Firstly, to evaluate the state mean $\mu_{a_{n},1}$ , three approximations are used: (S1) the prior PDF $p_{0}(a_{n})$ is constant over the connected region $I_{a_{n}}(z_{n})$ ; (S2) the nonlinear function h(⋅) can be locally linearized; and (S3) the measurement noise satisfies uniform distribution $e_{n}\sim U_{I_{e_{n}}}$ in the connected region $I_{e_{n}}$ . According to S2, the nonlinear measurement Equation (2) can be approximated as a linear estimator of $z_{n}$ , ${\hat{z}}_{n}$ such that:

(12) $${\hat{z}}_{n}=\hbar_{n}a_{n}+d_{n}$$

where $\hbar_{n}$ denotes a linear measurement matrix, and $d_{n}$ denotes a noise vector, which are derived by minimizing the objective function defined as follows:

(13) $$\left\{\hbar_{n},d_{n}\}=\arg\min E({\tau_{n}}^{T}\tau_{n}\right)$$

where $\tau_{n}$ is the linearization error, $\tau_{n}=z_{n}-{\hat{z}}_{n}$ .

Substituting $\tau_{n}$ into (13), and setting the partial derivative of the objective function with $d_{n}$ to zero,

(14) $$\begin{array}{l} (-2)E(z_{n}-\hbar_{n}a_{n}-d_{n})=0\\ \Leftrightarrow d_{n}={\bar{z}}_{n}-\hbar_{n}{\bar{a}}_{n} \end{array}$$

where ${\bar{a}}_{n}=E(x_{n})$ and ${\bar{z}}_{n}=E(z_{n})$ . Substituting $d_{n}$ into (13)

$$\tau^{T}\tau ={\left[(z_{n}-{\bar{z}}_{n})-\hbar_{n}(a_{n}-{\bar{a}}_{n})\right]}^{T}[(z_{n}-{\bar{z}}_{n})-\hbar_{n}(x_{n}-{\bar{x}}_{n})]$$

Then, setting the gradient with respect to $\hbar_{n}$ to zero,

(15) $$(-2)E\{[(z_{n}-{\bar{z}}_{n})-\hbar_{n}(a_{n}-{\bar{a}}_{n})][z_{n}-{\bar{z}}_{n}])\}=0$$

Solving (15) for $\hbar_{n}$ , we obtain:

(16) $$\hbar_{n}=P_{a_{n}z_{n}}^{T}P_{a_{n}a_{n}}^{-1}$$

where $P_{a_{n}a_{n}}=E[(a_{n}-{\bar{a}}_{n}){\left(a_{n}-{\bar{a}}_{n}\right)}^{T}]$ , $P_{a_{n}z_{n}}=E[(a_{n}-{\bar{a}}_{n}){\left(z_{n}-{\bar{z}}_{n}\right)}^{T}]$ . According to the measurement equation (12), the maximum likelihood position ${\hat{a}}_{n}(z_{n})$ of target state $a_{n}$ can be estimated as follows:

(17) $$\hat{a}(z_{n})=\hbar_{n}\cdot (z_{n}-d_{n})$$

If the maximum likelihood estimate ${\hat{a}}_{n}(z_{n})$ is used to replace the estimated mean $\mu_{a_{n},1}$ , the performance of the proposed algorithm will be improved because the current measurement information is incorporated. However, it cannot solve the uncertainty in motion models. In particular, when the target speed or the measurement sampling interval is large, the tracking performance degrades.

More recently, sophisticated techniques have been based on the target motion characteristics [6], which have been proposed to address the challenges in motion modeling. In their proposed method, three target characteristics, such as actual target speed v , time interval T of measurement and course angle θ of the target, are considered to improve the tracking performance. In (18), the relationship between three target characteristics and the state predicted error is given:

(18) $$\begin{array}{ll} \nabla \sigma & =\sqrt{{\left(T\cdot v\right)}^{2}+{\left(T\cdot v_{n}\right)}^{2}-2T^{2}v\cdot v_{n}\cos(\nabla \theta )}\\ & =T\cdot \sqrt{{\left(v\right)}^{2}+{\left(v_{n}\right)}^{2}-2v\cdot v_{n}\cos(\nabla \theta )} \end{array}$$

where $v_{n}$ denotes the current estimated velocity, ∇θ denotes the estimated error of course angle, and v denotes the actual target velocity. Supposing $\nabla v=v-v_{n}$ , we can obtain

(19) $$\begin{array}{ll} \nabla \sigma & =T\cdot \sqrt{{\left(v\right)}^{2}+{\left(v_{n}\right)}^{2}-2v\cdot v_{n}\cos(\nabla \theta )}\\ & =T\cdot \sqrt{\nabla {v_{n}}^{2}+2v\cdot \left(v-\nabla v_{n}\right)\left(1-\cos\left(\nabla \theta \right)\right)} \end{array}$$

From Equation (19), we can find that the predicted error ∇σ increases monotonically with the increase of the parameters ( $v_{n}$ , T , ∇θ ). In fact, when $\nabla v_{n}>50m/s$ and T>20s , the predicted error ∇σ will larger than 1000 m. It shows that the predicted error becomes a major reason for the performance degradation. On the other hand, when the actual target speed is relatively small, the prediction error caused by T or ∇θ is smaller than the measurement error. Therefore, to improve the performance of mean estimate $\mu_{a_{n},1}$ , the maximum likelihood estimate ${\hat{a}}_{n}(z_{n})$ is considered as the latest observation, and the modified maximum likelihood estimates that can incorporate current target characteristics is defined as follows:

(20) $$\hat{\phi }(z_{n})=\mu_{a_{n},0}+K_{n}(\hat{a}(z_{n})-\hbar_{n}\mu_{a_{n},0})$$

$$K_{n}=\left(T^{2}\cdot v^{2}\cdot \sigma_{v}^{2}\left(n\right)\right)/\left(\lambda \cdot \sigma_{e}^{2}\left(k\right)+T^{2}\cdot v^{2}\cdot \sigma_{v}^{2}\left(n\right)\right)$$

where λ is a constant, $\sigma_{e}^{2}\left(n\right)$ denotes the variance of measurement noise, and $\sigma_{v}^{2}\left(n\right)$ denotes the innovation variance. According to (20) and (16), the mean $\mu_{a_{n},1}$ and the variance $\Sigma_{a_{n},1}$ in (10) can be approximated as:

(21) $$\mu_{a_{n},1}=\hat{\phi }(z_{n}),\Sigma_{a_{n},1}=P_{a_{n}a_{n}}$$

Finally, the modified prior PDF $p_{1}(\cdot )$ can be approximated as a Gaussian probability density function $p_{1}(\cdot )\approx N(x_{n},{\hat{x}}_{p_{1},n},P_{p_{1},n})$ .

### 2.3. Summary of the Proposed Algorithm

**Figure 1:**
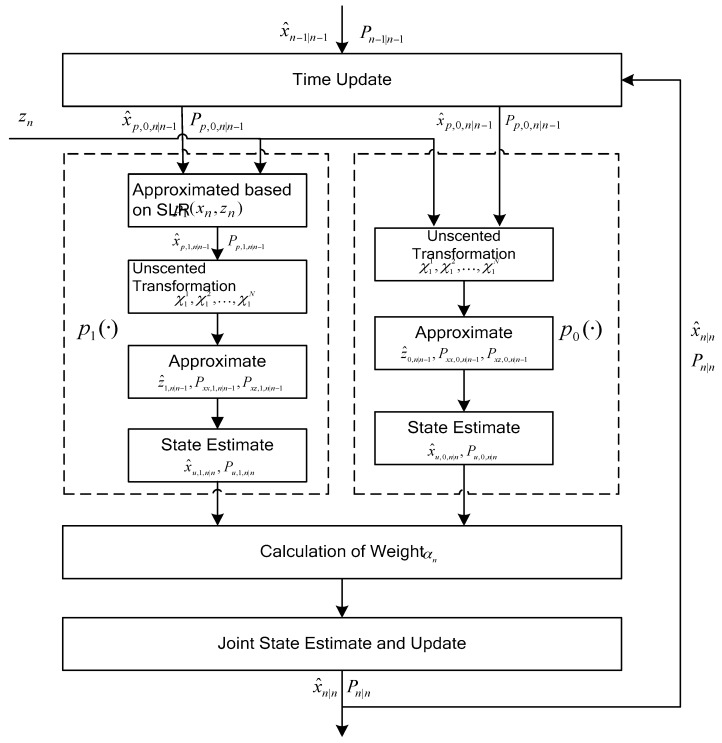
Auxiliary truncated unscented Kalman filtering.

According to the descriptions above, in order to describe clearly the proposed algorithm, the diagram of the ATUKF is shown in Figure 1. In Figure 1, it is shown that one cycle of the ATUKF algorithm consists of the following steps: (A) time update (predicted by using Kalman filtering); (B) the measurement updates based on the prior $p_{0}\left(\cdot \right)$ and the modified prior $p_{1}\left(\cdot \right)$ ; and (C) weight calculation and the joint state update. According to the derived results mentioned above and Figure 1, the detailed information of the new ATUKF algorithm can be summarized as follows.

#### Algorithm: Auxiliary Truncated Unscented Kalman Filtering (ATUKF)

**ATUKF—Update based on the prior probability density function (PDF)** $p_{0}(\cdot )$

1. Obtain $N=2n_{x}+1$ sigma points $\chi_{0}^{1},\chi_{0}^{2}...,\chi_{0}^{N}$ and the corresponding associated weights $w_{1},w_{2},...,w_{N}$ using unscented transform (UT ) based on the mean ${\hat{x}}_{n-1|n-1}$ and covariance $P_{n-1|n-1}$ of the posterior PDF $p_{0}(x_{n}|x_{n-1},z_{1:n-1},r_{1;n-1})$ , where $n_{x}$ denotes the dimension of state x . The predicted sigma points can be obtained by the nonlinear state function f(⋅) :
(22) $$\chi_{0,n|n-1}^{i}=f(\chi_{0}^{i}),\;i=1,2,\ldots ,N$$
2. Approximate the mean and covariance of the state-predicted prior PDF $p_{0}(x_{n}|x_{n-1},z_{1:n-1})$
(23) $${\hat{x}}_{p,0,n|n-1}=\sum_{i=1}^{N}w_{i}\chi_{0,n|n-1}^{i}$$
(24) $$P_{p,0,n|n-1}=Q_{n}+\sum_{i=1}^{N}w_{i}(\chi_{0,n|n-1}^{i}-{\hat{x}}_{p,0,n|n-1}){\left(\chi_{0,n|n-1}^{i}-{\hat{x}}_{p,0,n|n-1}\right)}^{T}$$
3. Compute the predicted measurement ${\hat{z}}_{0,n|n-1}$ based on the nonlinear measurement function h(⋅) :
(25) $$z_{0,n|n-1}^{i}=h(\chi_{0,n|n-1}^{i}),\;i=1,2,\ldots ,N$$
(26) $${\hat{z}}_{0,n|n-1}=\sum_{i=1}^{N}w_{i}z_{0,n|n-1}^{j}$$
4. The cross-covariance, innovation covariance and error covariance are estimated as follows:
(27) $$P_{xz,0,n|n-1}=\sum_{i=1}^{N}w_{i}(\chi_{0,n|n-1}^{i}-{\hat{x}}_{p,0,n|n-1}){\left(z_{0,n|n-1}^{i}-{\hat{z}}_{0,n|n-1}\right)}^{T},\;(Cross\;\mathrm{covariance})$$
(28) $$P_{zz,0,n|n-1}=R_{n}+\sum_{i=1}^{N}w_{i}(z_{0,n|n-1}^{i}-{\hat{z}}_{0,n|n-1}){\left(z_{0,n|n-1}^{i}-{\hat{z}}_{0,n|n-1}\right)}^{T},\;(Innovation\;\mathrm{covariance})$$
(29) $$P_{xx,0,n|n-1}=\sum_{i=1}^{N}w_{i}(\chi_{0,n|n-1}^{i}-{\hat{x}}_{p,0,n|n-1}){\left(\chi_{0,n|n-1}^{i}-{\hat{x}}_{p,0,n|n-1}\right)}^{T},\;(Error\;\mathrm{covariance})$$
5. Estimate the mean ${\hat{x}}_{u,0,n|n}$ and covariance $P_{u,0,n|n}$ using (30) and (31):
(30) $${\hat{x}}_{u,0,n|n}={\hat{x}}_{p,0,n|n-1}+P_{xz,0,n|n-1}P_{zz,0,n|n-1}^{-1}(z_{n}-{\hat{z}}_{0,n|n-1})$$
(31) $$P_{u,0,n|n}=P_{p,0,n|n-1}-P_{xz,0,n|n-1}P_{zz,0,n|-1}^{-1}P_{xz,0,n|n-1}^{T}$$

**ATUKF—Update based on the modified prior PDF** $p_{1}(\cdot )$

1. Calculation of the mean ${\hat{x}}_{p,1,n|n-1}$ and covariance $P_{p,1,n|n-1}$ of the prior $p_{1}(\cdot )$
  According to (14) and (16) in Section 2.2, the linear regression coefficients $\hbar_{n}$ and $d_{n}$ can be approximately computed by using Equations (27–29). The mean ${\hat{x}}_{p,1,n|n-1}$ and covariance $P_{p,1,n|n-1}$ of $p_{1}(\cdot )$ can be approximately estimated by (10) and (11), respectively.
2. Draw N new sigma points $\chi_{1,n|n-1}^{1},\chi_{1,n|n-1}^{2},\ldots ,\chi_{1,n|n-1}^{N}$ with the associated weights $w_{1},w_{2},......w_{N}$ by using the UT based on the mean ${\hat{x}}_{p,1,n|n-1}$ and covariance $P_{p,1,n|n-1}$ . The predicted measurements of new sigma points are estimated as follows:
(32) $$z_{1,n|n-1}^{i}=h(\chi_{1,n|n-1}^{i})$$
3. Calculation of ${\hat{z}}_{1,n|n-1}$ , $P_{zz1,n|n-1}$ and $P_{xz1,n|n-1}$
(33) $${\hat{z}}_{1,n|n-1}=\sum_{i=1}^{N}w_{i}z_{1,n|n-1}^{j}$$
(34) $$P_{zz,1,n|n-1}=R_{n}+\sum_{i=1}^{N}w_{i}(z_{1,n|n-1}^{i}-{\hat{z}}_{1,n|n-1}){\left(z_{1,n|n-1}^{i}-{\hat{z}}_{1,n|n-1}\right)}^{T},\;(\mathrm{Innovation}\;\mathrm{covariance})$$
(35) $$P_{xz,1,n|n-1}=\sum_{i=1}^{N}w_{i}(\chi_{1,n|n-1}^{i}-{\hat{x}}_{p,1,n|n-1}){\left(z_{1,n|n-1}^{i}-{\hat{z}}_{1,n|n-1}\right)}^{T},\;(\mathrm{Cross}\;\mathrm{covariance})$$
4. Estimate the mean ${\hat{x}}_{u,1,n|n}$ and covariance $P_{u,1,n|n}$ using (36) and (37):
(36) $${\hat{x}}_{u,1,n|n}={\hat{x}}_{p,1,n|n-1}+P_{xz,1,n|n-1}P_{zz,1,n|n-1}^{-1}(z_{n}-{\hat{z}}_{1,n|n-1})$$
(37) $$P_{u,1,n|n}=P_{p,1,n|n}-P_{xz,1,n|n-1}P_{zz,1,n|n-1}^{-1}P_{xz,1,n|n-1}^{T}$$

**ATUKF—Jointly update**

1. Calculate the parameter $\alpha_{n}$ using (38) and (39)
(38) $$\mu_{1}({\hat{x}}_{u,i,n})=1/\sqrt{\left|P_{u,i,n}\right|}\cdot \exp(\left(z_{n}-h_{n}({\hat{x}}_{u,i,n})\right)/2),i=0,1$$
(39) $$\alpha_{n}=\mu_{1}({\hat{x}}_{u,1,n})/\left(\mu_{0}({\hat{x}}_{u,0,n})+\mu_{1}({\hat{x}}_{u,1,n})\right)$$
2. Approximate the mean ${\hat{x}}_{n}$ and covariance $P_{n}$ of the posterior PDF $p\left(x_{n}|z_{1:n}\right)$ using (23) and (24).
(40) $${\hat{x}}_{n|n}=\alpha_{n}\cdot {\hat{x}}_{u,1,n|n}+(1-\alpha_{n})\cdot {\hat{x}}_{u,0,n|n}$$
(41) $$P_{n|n}=\alpha_{n}\cdot \left[P_{u,1,n|n}+({\hat{x}}_{u,1,n|n}-\hat{x}{)}_{n|n}{({\hat{x}}_{u,1,n|n}-\hat{x}{)}_{n|n}}^{T}\right]+(1-\alpha_{n})\cdot \left[P_{u,0,n|n}+({\hat{x}}_{u,0,n|n}-{\hat{x}}_{n|n}){\left({\hat{x}}_{u,0,n|n}-{\hat{x}}_{n|n}\right)}^{T}\right]$$

## 3. Simulation Results

In this section, to evaluate the tracking performance of the ATUKF algorithm, two examples are employed. In Section 3.1, the univariate nonstationary growth model (UNGM) is discussed [11]. In Section 3.2, a bearings-only maneuvering tracking scenario [24], interested in defense applications, is discussed. In the first case, the EKF, UKF, the quadrature Kalman filtering (QKF) [25], the mixture truncated unscented Kalman filter (MTUKF, with three mixture components) [12] and particle filtering(PF) are utilized. In the second case, the TQKF, interacting multiple model extended Kalman filtering(IIMMEKF) and the interacting multiple model Rao–Blackwellized particle filter (IMMRBPF) are employed. In all the experiments, each simulation has been repeatedly performed 100 times.

### 3.1. Univariate Nonstationary Growth Model (UNGM)

In this section, due to the highly nonlinearity and non-stationarity of dynamic system, the univariate nonstationary growth model is considered. The discrete time system of this model can be written as:

(42) $$x_{n}=\alpha x_{n-1}+\beta \frac{x_{n-1}}{1+x_{n-1}^{2}}+\gamma \cos\left(1.2\left(n-1\right)\right)+m_{n}$$

(43) $$z_{n}=\left\{ \begin{array}{l} \varphi_{2}x_{n}^{2}+e_{n}\;n\leq 30\\ \varphi_{1}x_{n}^{3}-2+e_{n}\;n>30 \end{array}\right.$$

where the process noise $m_{n}$ is satisfied with Gaussian distribution with zero mean and variance one, and $e_{n}$ is satisfied with Gaussian distribution with zero mean and variance 0.01 . α=0.5 , β=25 , γ=8 , $\varphi_{1}=0.2$ and $\varphi_{2}=0.05$ are known constants. In each Monte Carlo simulation, we assume that the initial distribution of state $x_{0}$ is uniform distribution in the interval [0 1]. The number of particles is 1000.

**Figure 2:**
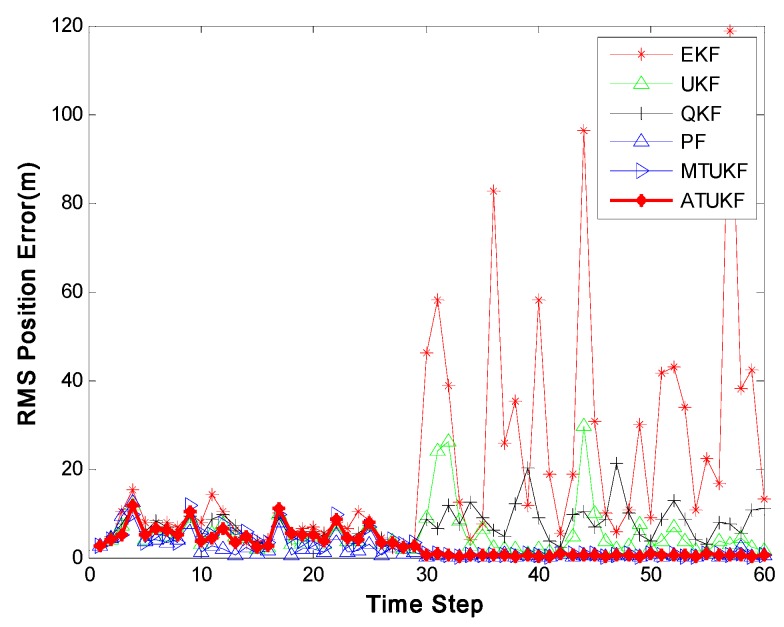
Root-mean-square (RMS) position errors of the extended Kalman filter (EKF), unscented Kalman filter (UKF), quadrature Kalman filtering (QKF), PF, mixture truncated unscented Kalman filter (MTUKF) and auxiliary truncated unscented Kalman filtering (ATUKF).

**Figure 3:**
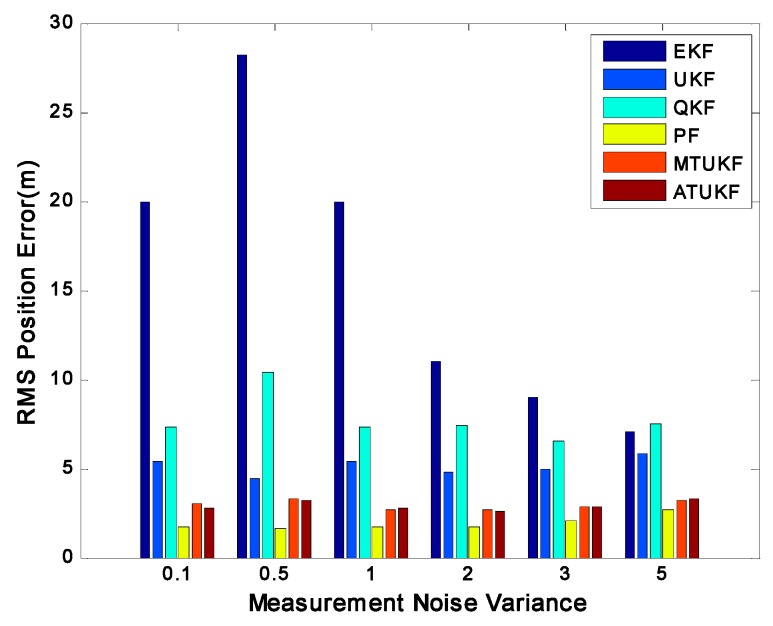
RMS position error for different noise variances.

Figure 2 shows the root-mean-square position errors (RMSE) of the EKF, UKF, QKF, PF, MTUKF and ATUKF. It is obvious from Figure 2 that the performance of the ATUKF is much better than that of the EKF, UKF and QKF. In this case, the performance of the EKF is the poorest. A reason for the poor performance of the EKF, UKF and QKF is the increase of the approximate error arising from the high nonlinearity and the non-stationarity of the dynamic system. Figure 3 shows the RMS position errors of all filters with different noise variance $\sigma_{e_{n}}\sim [0.1\;5]$ . From Figure 3, it is seen that whenever the measurement is informative ( $\sigma_{e_{n}}<1$ ) or the measurement is uninformative ( $\sigma_{e_{n}}>1$ ), the ATUKF is robust in all situations, its performance is similar to the MTUKF’s, and it is very close to that of the PF. Moreover, among the EKF, UKF and QKF, the EKF has the poorest performance. In particular, when the measurement is very informative ( $\sigma_{e_{n}}<1$ ), the EKF yields a divergent estimate.

Table 1 shows the computation time statistics for all algorithms. In this case, all the experiments are performed by using MATLAB programming on Intel-Core(TM)-i2-4030U processor (1.9 GHz) based on the Windows platform. It can be seen from Table 1 that the computational load of the PF is the largest than these of other filters, such as the EKF, UKF, QKF, MTUKF and ATUKF. The ATUKF is very close to the QKF. Furthermore, the computation time for the ATUKF is much lower than the MTUKF. The main reason is that the MTUKF approximates the posterior PDF as a Gaussian mixture, and it makes the computational burden increase.

### 3.2. Bearings-Only Maneuvering Tracking (BOT) Scenario

**Figure 4:**
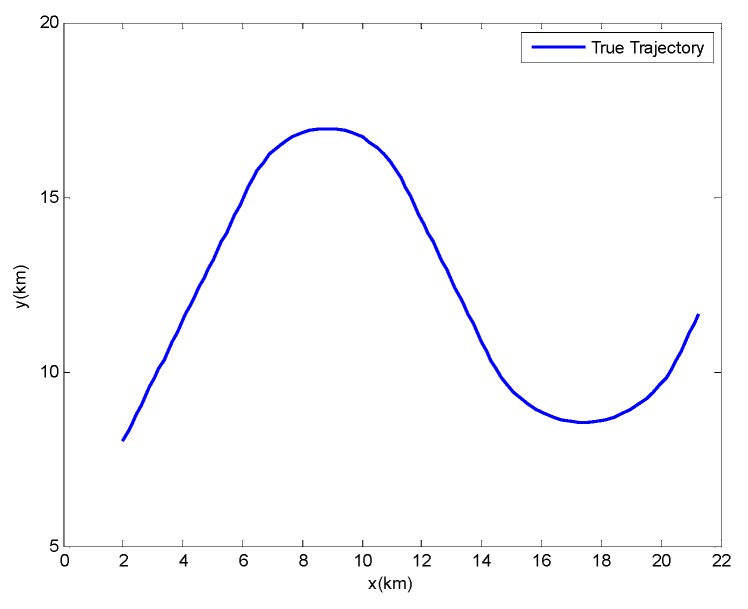
True Trajectory.

In this scenario, the target makes two circular turns with rectilinear segments connecting them. Figure 4 shows the true target trajectory. The speed modulus is kept constant throughout (0.3 km/s) . The initial position is (2 km, 8 km, 1 km) , and the initial velocity is (0.15 km/s, 0.26 km/s, 0.0 km/s) . The segments are defined as follows:

First segment. Rectilinear flight until the plane is at (6.35 km, 15.53 km, 1 km) (from *t* = 0 s to *t* = 30 s).

Second segment. Circular turn with turn rate $6^{o}/s$ (from *t* = 31 s to *t* = 50 s).

Third segment. Rectilinear flight until the plane is at (14.31 km, 10.33 km, 1 km) (from *t* = 51 s to *t* = 70 s).

Fourth segment. Circular turn with turn rate $4.8^{o}/s$ (from *t* = 71 s to *t* = 95 s).

Fifth segment. Rectilinear flight until the plane is at (21.26 km, 11.63 km, 1 km) (from *t* = 96s to *t* = 100 s).

The motion model of target is defined as follows:

(44) $$x_{n}^{s}=F^{s}x_{n-1}^{s}+m_{n}^{s}$$

where $x_{n}^{s}=(x,y,z,x^{\prime },y^{\prime },z^{\prime })$ denotes the target state vector under model x , x , y and z denote the target position coordinates, $x^{\prime }$ , $y^{\prime }$ and $z^{\prime }$ are the target speed in x , y and z directions, respectively, $F^{s}$ denotes the transition matrix, and s∈{1,2,…,M} denotes the target model index. In the maneuvering target tracking scenario, only a constant velocity model is used for the ATUKF algorithm and TQKF algorithm. In the IMMEKF and IMMRBPF, there are both clockwise- and counterclockwise-coordinated turn models that are used to simulate the target maneuvering. The details of three target motion models are defined as follows:

**Model 1:** Constant Velocity Motion

The state transition matrix and the process noise covariance matrix are defined by:

(45) $$F^{1}=\left[\begin{array}{cccccc} 1 & 0 & 0 & T & 0 & 0\\ 0 & 1 & 0 & 0 & T & 0\\ 0 & 0 & 1 & 0 & 0 & T\\ 0 & 0 & 0 & 1 & 0 & 0\\ 0 & 0 & 0 & 0 & 1 & 0\\ 0 & 0 & 0 & 0 & 0 & 1 \end{array}\right]$$

(46) $$Q^{1}=\left[\begin{array}{cccccc} \frac{1}{4}T^{4} & 0 & 0 & \frac{1}{2}T^{3} & 0 & 0\\ 0 & \frac{1}{4}T^{4} & 0 & 0 & \frac{1}{2}T^{3} & 0\\ 0 & 0 & \frac{1}{4}T^{4} & 0 & 0 & \frac{1}{2}T^{3}\\ \frac{1}{2}T^{3} & 0 & 0 & T^{2} & 0 & 0\\ 0 & \frac{1}{2}T^{3} & 0 & 0 & T^{2} & 0\\ 0 & 0 & \frac{1}{2}T^{3} & 0 & 0 & T^{2} \end{array}\right]\cdot \sigma_{n}^{2}$$

where T denotes the sampling interval, in this paper, T is set to 1.

**Model 2:** Constant Turn Motion

The state transition matrix is:

(47) $$F^{2}=\left[\begin{array}{cccccc} 1 & 0 & 0 & \frac{\sin(w)}{w} & \frac{\cos(w)-1}{w} & 0\\ 0 & 1 & 0 & \frac{1-\cos(w)}{w} & \frac{\sin(w)}{w} & 0\\ 0 & 0 & 1 & 0 & 0 & 1\\ 0 & 0 & 0 & \cos(w) & -\sin(w) & 0\\ 0 & 0 & 0 & \sin(w) & \cos(w) & 0\\ 0 & 0 & 0 & 0 & 0 & 1 \end{array}\right]$$

where w is a constant angular rate. In this paper, w is set to 0.0175 . The process noise covariance matrix $Q^{2}$ is the same as in Model 1.

**Model 3:** w>0 describes a clockwise turn, and Model 3 is its natural counterpart for a counterclockwise turn w<0 .

Two passive sensors are deployed in (0, −5 km, 0) and (0, 5 km, 0) respectively. Using the detection fusion architecture [24], the azimuth and elevation angles of aircraft, $\alpha_{i}$ and $\beta_{i}$ respectively, measured by sensor i , are transmitted to the fusion node. The measurement function is written as:

(48) $$h\left(x_{n}\right)=\left(\begin{array}{l} \alpha_{i}\\ \beta_{i} \end{array}\right)=\left(\begin{array}{l} \arctan\left(\frac{y-s_{i,y}}{x-s_{i,x}}\right)\\ \arctan\left(\frac{z-s_{i,z}}{\sqrt{{\left(x-s_{i,x}\right)}^{2}+{\left(y-s_{i,y}\right)}^{2}}}\right) \end{array}\right)$$

where $(s_{i,x},s_{i,y},s_{i,z}),i=1,2$ denote the positions of the stationary sensors. The measurement covariance can be defined as $R_{n}=\left[\begin{array}{cc} 1 & 0\\ 0 & 1 \end{array}\right]\sigma_{e_{n}}^{2}$ .

The real initial position of the target is (2 km, 8 km, 1 km) , and the initial velocity is (0.15 km/s, 0.26 km/s, 0.0 km/s) . The prior PDF of state $x_{0}$ is assumed to be $x_{0}\sim N\left({\hat{x}}_{0|0},{\hat{P}}_{0|0}\right)$ , where ${\hat{x}}_{0|0}={\left[2.1\;\mathrm{km}\;0{0.12\;\mathrm{kms}}^{-1}\;7.95\;\mathrm{km}\;{0.23\;\mathrm{kms}}^{-1}\;{0.95\;\mathrm{km}\;0\;\mathrm{kms}}^{-1}\right]}^{T}$ , ${\hat{P}}_{0|0}=diag{\left[0.144\;\mathrm{km}{\;}^{2}\;0.02^{2}{\;\mathrm{km}}^{2}s^{-2}\;{0.144\;\mathrm{km}}^{2}\;0.02.^{2}\;{\mathrm{km}}^{2}s^{-2}\;0\;0\right]}^{T}$ . The standard deviation of the process and the measurement noise are set to $\sigma_{m_{n}}=0.01$ and $\sigma_{e_{n}}=0.001$ , and these noises are zero-mean Gaussian-distributed and independent. The number of particles is set to 200.

**Figure 5:**
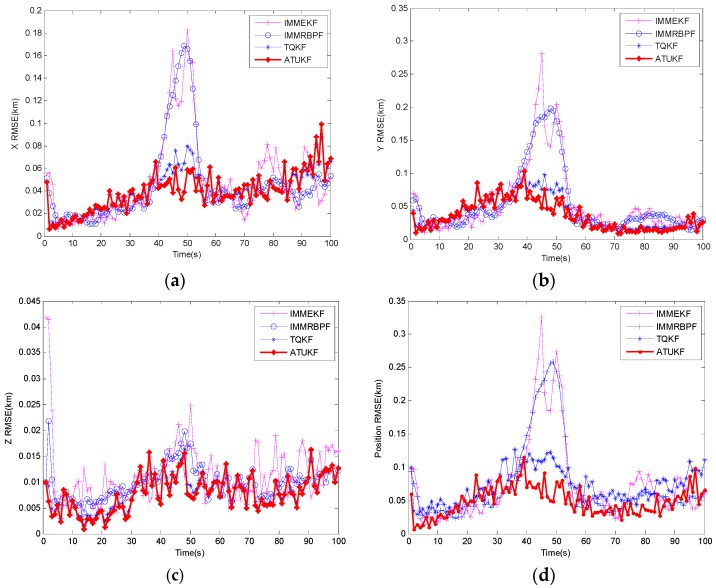
Root-mean-square error (RMSE) of the ATUKF, IMMEKF and IMMRBPF. (**a**) X RMSE; (**b**) Y RMSE; (**c**) Z RMSE; (**d**) position RMSE.

Figure 5 shows the X RMSE, Y RMSE, Z RMSE and position RMSE of the ATUKF compared with IMMEKF, IMMRBPF and TQKF. From Figure 5, we can see that the RMSE of all algorithms abruptly increased from 30 s to 50 s, which is mainly due to the increase of the target predicted errors caused by the target maneuvering. However, in Figure 5a,c,d, it is shown that the performance of the ATUKF algorithm has outperformed the IMMEKF, IMMRBPF and TQKF. A key reason is that the proposed algorithm can incorporate the target characteristic information and current measurement information into the prior PDF, which can effectively degrade the variance of errors caused due to the maneuvering of the target. Moreover, because the flight height of target remained unchanged, from Figure 5c, the Z RMSE of all algorithms is very close.

Finally, the computation time statistics for all algorithms are given in Table 2. In this case, all the experiments are performed by using MATLAB programming on an Intel-Core(TM)-i2-4030U processor (1.9 GHz) based on the Windows platform. In Table 2, it is shown that the computational load of the IMMRBPF is much higher than these of the IMMEKF, TQKF and ATUKF. More importantly, the ATUKF requires much less of a computation time than the TQKF. However, the computational load of the ATUKF is nearly two times higher than that of the IMMEKF.

## 4. Conclusions

In this paper, we presented a bearings-only target tracking algorithm based on an auxiliary truncated unscented Kalman filtering (ATUKF) algorithm. Unlike the truncated unscented Kalman filtering, in the proposed algorithm, several target characteristics were introduced to construct the modified prior PDF, and the statistical linear regression was used to linearize the nonlinear non-bijective measurement function by using the sigma points. Moreover, we have developed a practical algorithm for a bearings-only target tracking system. Finally, in the simulation results, compared with the EKF, UKF, the quadrature Kalman filtering (QKF), the mixture truncated unscented Kalman filter (MTUKF) and the particle filter (PF), the ATUKF exhibits better performance. For the second case, compared with the IMMEKF algorithm, the IMMRBPF algorithm and the TQKF algorithm, the ATUKF algorithm not only improves the performance of the tracker, but significantly reduces the computation time.

## Figures and Tables

**Table 1:** Comparison of the computation times of different filtering algorithms (s). UNGM: univariate nonstationary growth model.

Case	EKF	UKF	QKF	PF	MTUKF(3)	ATUKF
UNGM	1.102	6.650	15.264	522.519	43.142	16.240

**Table 2:** Computation times for all algorithms (s). BOT: Bearings-only maneuvering tracking.

Case	IMMEKF	IMMRBPF	TQKF	ATUKF
BOT	0.074	14.493	0.553	0.150
